# Economic evaluation of a multi-strategy intervention that improves school-based physical activity policy implementation

**DOI:** 10.1186/s13012-022-01215-6

**Published:** 2022-06-28

**Authors:** Cassandra Lane, Nicole Nathan, Penny Reeves, Rachel Sutherland, Luke Wolfenden, Adam Shoesmith, Alix Hall

**Affiliations:** 1 Hunter New England Population Health, Hunter New England Area Health Service, Newcastle, NSW Australia; 2 School of Medicine and Public Health, The University of Newcastle, Callaghan, NSW Australia; 3 Priority Research Centre for Health Behaviour, The University of Newcastle, Callaghan, NSW Australia; 4 Hunter Medical Research Institute (HMRI), New Lambton, NSW Australia

**Keywords:** Economic evaluation, Implementation strategy, Physical activity, Schools, Childhood obesity prevention

## Abstract

**Background:**

Internationally, government policies mandating schools to provide students with opportunities to participate in physical activity are poorly implemented. The multi-component *Physically Active Children in Education* (PACE) intervention effectively assists schools to implement one such policy. We evaluated the value of investment by health service providers tasked with intervention delivery, and explored where adaptations might be targeted to reduce program costs for scale-up.

**Methods:**

A prospective trial-based economic evaluation of an implementation intervention in 61 primary schools in New South Wales (NSW), Australia. Schools were randomised to the PACE intervention or a wait-list control. PACE strategies included centralised technical assistance, ongoing consultation, principal's mandated change, identifying and preparing in-school champions, educational outreach visits, and provision of educational materials and equipment. Effectiveness was measured as the mean weekly minutes of physical activity implemented by classroom teachers, recorded in a daily log book at baseline and 12-month follow-up. Delivery costs (reported in $AUD, 2018) were evaluated from a public finance perspective. Cost data were used to calculate: total intervention cost, cost per strategy and incremental cost (overall across all schools and as an average per school). Incremental cost-effectiveness ratios (ICERs) were calculated as the incremental cost of delivering PACE divided by the estimated intervention effect.

**Results:**

PACE cost the health service provider a total of $35,692 (95% uncertainty interval [UI] $32,411, $38,331) to deliver; an average cost per school of $1151 (95%UI $1046, $1236). Training in-school champions was the largest contributor: $19,437 total; $627 ($0 to $648) average per school. Educational outreach was the second largest contributor: $4992 total; $161 ($0 to $528) average per school. The ICER was $29 (95%UI $17, $64) for every additional minute of weekly physical activity implemented per school.

**Conclusion:**

PACE is a potentially cost-effective intervention for increasing schools implementation of a policy mandate. The investment required by the health service provider makes use of existing funding and infrastructure; the additional cost to assist schools to implement the policy is likely not that much. PACE strategies may be adapted to substantially improve delivery costs.

**Trial registration:**

Australia New Zealand Clinical Trials Registry ACTRN12617001265369; Prospectively registered 1st September 2017 https://www.anzctr.org.au/Trial/Registration/TrialReview.aspx?id=373520

Contributions to the literature

- Few economic evaluations of implementation interventions in the school setting have been published, and none have explored physical activity policy implementation, resulting in little understanding of the investment required to achieve implementation of an evidence-based practice.
- This study is one of very few to assess cost-effectiveness in terms of an implementation outcome; thus, building an evidence base for future cost-effectiveness analyses of implementation interventions in schools and similar settings.
- These findings contribute to several identified evidence gaps and provide important financial information that may assist researchers and policy-makers to make educated decisions regarding policy implementation strategies.

## Background

The importance of physical activity in childhood is well established [1–3], yet few children internationally are meeting levels needed to achieve health benefits [4]. A 2015 study of objective data consolidated from 20 studies conducted across ten countries showed the proportion of children meeting the World Health Organization (WHO) physical activity guidelines to be as low as 9% for boys and 1.9% for girls [5]. This is concerning as insufficient levels of physical activity is associated with the development of chronic diseases such as coronary heart disease, type 2 diabetes, and several types of cancer [6]. Physical inactivity is also the fourth leading cause of death worldwide [7] and places a considerable economic burden on society, due to impacts such as workforce productivity losses, absenteeism, presenteeism [8]; and healthcare-related costs [9] associated with the mortality and morbidity attributable to physical inactivity. For example, the cost of physical inactivity in 2013 was conservatively estimated at $67.5 billion globally as a result of direct health-care costs ($53.8 billion) and indirect costs such as productivity loss ($13.7 billion) [9].

Creating school environments supportive of physical activity has been identified as a priority by the WHO [10], researchers [11, 12], and governments internationally [13–15]. School-based interventions can be effective in improving children’s physical activity levels [3, 16]; particularly interventions that employ policies mandating specific time that schools are to provide students with opportunities to be active [12, 17]*.* Such interventions may also be cost-effective [18–22]. For example, a 2021 micro-simulated economic evaluation of Canadian school health programs found that programs providing students with greater time to be physically active (e.g. through increased duration of physical education [PE] and increased physical activity as part of comprehensive school health programs) were cost-effective and offered substantial health care cost-savings (Canadian dollar [CAD]$484 and $824 for each $100 spent per student), equating to > 450% return on investment by avoiding the treatment and management of chronic diseases over the life course [21].

Governments worldwide, including Australia [23], China [24], Denmark [25], England [26] as well as several jurisdictions in Canada [27] and the USA [28], have developed policies for schools to provide students with a minimum amount of daily or weekly physical activity. Unfortunately implementation of such policies is often poor [27–35] with studies showing as few as 20–30% of schools comply with mandatory policies. If the potential benefits of these school physical activity policies are to be realised, their population-wide implementation is required. There are, however, few rigorously evaluated interventions assessing the effectiveness of strategies to increase schools’ implementation of physical activity policies [36]. To address this evidence gap we recently undertook a randomised controlled trial (RCT) in 61 Australian primary schools to determine the effectiveness of a 12-month multi-strategy intervention to support schools’ implementation of a mandatory state physical activity policy [37]. Strategies consisted of centralised technical assistance and ongoing consultation/coaching from an external support officer; principals mandating for change via discourse, school-wide promotion, and school-policy development; identifying in-school champions and preparing them with a 1-day training workshop; educational outreach visits for all school staff delivered by an external support officer; and provision of educational materials and a physical activity equipment pack. This is compared to schools randomly allocated to the control condition, who received usual care to support their implementation of the policy. Usual care includes access to general resources and information (accessible to all schools) and reactive support from health service staff upon request by the school. Intervention schools also had access to usual care, however the suite of PACE strategies were designed to replace the reactive support of usual care. At 12-month follow-up, teachers at intervention schools recorded a significantly greater increase in their implementation of mean weekly minutes of physical activity than teachers at control schools by approximately 44.2 min (95% CI 32.8 to 55.7; *p* < 0.001) [37].

Following the research-practice continuum [38], the next logical step is to scale-up our effective implementation intervention to reach more schools, thus maximising population health impacts [39]. However, for an intervention to be considered by policy-makers for broader dissemination it needs to be both effective and cost-effective [40, 41]. Cost-effectiveness analysis (CEA) is a form of economic evaluation that measures outcomes in terms of a naturally occurring, non-monetised health outcome of interest [42], for example reduction in body mass index (BMI) or life years saved. CEA findings serve as a point of reference for decision-makers to make informed choices on the allocation of scarce public health resources [40]. Cost information may also inform the feasibility of an intervention being successfully scaled-up and sustained [40, 43]. Finally, cost data can highlight the more costly intervention components for targeting adaptations to reduce intervention cost. Any cost-savings may improve the likelihood of investment in scale-up, particularly in low-resourced areas.

Unfortunately few studies of public health implementation interventions report on costs and/or cost-effectiveness [41]. In a 2019 systematic review of economic evaluations applied to public health implementation interventions, only 14 studies were identified over a 27-year span (1990–2017) [41]. Of these, nine were considered cost-effective (only one of which was performed in the school setting [44]) or had a positive cost-benefit ratio [41]; however cost-effectiveness estimates were scarce and the context for such claims were rarely provided. As a result, no broad conclusions regarding the value for money offered by implementation interventions applied to public health interventions were possible. Further economic evaluations of public health implementation interventions have emerged since this review [45–50]; however, the field of work remains small and studies are highly variable by topic, research design, setting and outcome measure(s). Cost evaluations of public health implementation interventions, especially via cost-effectiveness estimates, are needed to enhance understanding and ultimately to increase the uptake of evidence-based practices across settings [41, 43, 51, 52]. Given the established evidence base demonstrating the cost-effectiveness of increasing physical activity in schools [18–22], we sought to determine the efficiency of the additional investment required to increase policy implementation.

We conducted a trial-based economic evaluation of a multi-strategy intervention to support schools’ implementation of a mandatory state physical activity policy. In line with best practice [40, 41], the study aimed to:

- Evaluate the value of investment by health service providers tasked with intervention delivery, in order to provide valuable information for decision makers interested in scaling-up policy implementation; and
- Explore more costly intervention components where adaptations might be targeted to reduce program costs for delivery at scale, which is information sought-after by our research team to improve the intervention and increase likelihood of successful scale-up in other contexts.

## Methods

### Trial design, setting and sample

This economic study used data from the 2017–2019 ‘Physically Active Children in Education (PACE)’ cluster RCT undertaken in 61 primary schools from the Hunter New England region of New South Wales (NSW), Australia [37, 53]. Consenting, eligible schools were randomly assigned to receive PACE (*n* = 31) or a wait-list control (*n* = 30). Full details of the study methods [53] and intervention effects [37] are reported elsewhere. The trial was prospectively registered with the Australian New Zealand Clinical Trials Registry (ACTRN12617001265369). Ethics approval was received from the Hunter New England Human Research Ethics Committee (no. 06/07/26/4.04), The University of Newcastle Human Research Ethics Committee (no. H-2008-0343), and relevant school bodies.

### Economic study

We conducted a trial-based prospective economic evaluation of PACE from a public finance perspective including both the health service (base case) and schools (sensitivity analysis). The cost evaluation occurred over a 12-month period consistent with the duration of the delivery of the implementation intervention. Costs were reported in Australian dollars (AUD) using 2018 as the base-year value. The economic evaluation in the present study adheres to the Consolidated Health Economic Evaluation Reporting Standards (CHEERS) Statement [54]. The primary economic outcome was the incremental cost effectiveness ratio (ICER). This ratio is calculated as the incremental cost of the intervention (numerator) divided by the incremental primary trial outcome (denominator). In this trial, the outcome was measured as the number of additional minutes of weekly physical activity implemented by classroom teachers, per school, at 12-month follow-up. This is consistent with the primary outcome from the trial and appropriately focussed on the measurement of policy implementation as opposed to clinical outcomes, given this is an implementation trial [43]. In line with recommendations [55, 56], we used a cost-consequence analysis to accommodate the range of secondary outcomes measured in the trial; presenting cost and effects in a disaggregated format for interpretation by decision-makers. Moreover, as the secondary outcome measures reflected the individual components that made up the primary outcome of total physical activity (PE, energisers, sport and integrated lessons), a cost-consequence analysis may assist decision makers in determining whether greater focus may be provided to one or more type of physical activity to improve intervention efficiency [57].

### Comparator

In NSW, Local Health Districts (the health service) are currently funded approximately AUD$700 annually per school to provide schools with support to implement state-wide obesity prevention programs, including the Department of Education mandatory Sport and Physical Activity policy [58] (hereafter referred to as “the policy” and the focus of our implementation trial). This policy requires public schools to incorporate 150 min of moderate, with some vigorous physical activity across the school week for students in Kindergarten to Grade 10. This may include: PE, sport or other structured activities such as energisers (a 3–5-min structured classroom physical activity break) or integrated lessons (incorporating physical activity into other curricular subjects). In this economic analysis, the support that comprises usual care included access to general resources and information via a dedicated website [53] as well as reactive support, i.e. provided upon the request of individual schools via email, telephone or in-person contact and referral to supporting resources. This support is provided by health service personnel who are external to the school and education system. Despite this available support, implementation of the policy in NSW schools remains substandard and few schools seek support in this regards. The funding for usual practice covers activities to support a range of obesity prevention programs, including the policy of interest, which cannot be disentangled. As a result, we took a conservative approach for the base case analysis and assumed that all costs associated with the intervention were wholly incremental to usual care. This prevents overestimation for any claims of cost effectiveness.

### The PACE intervention

**Fig. 1:**
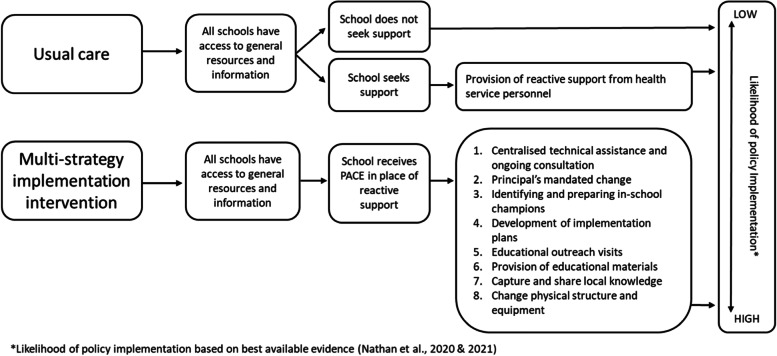
The progression of schools to implement the policy through either usual care or the suite of strategies (PACE) designed to replace the reactive support available in usual care. All schools have access to general resources and information. Following the usual care path: upon school request, project officers provide reactive support. Following the PACE path: schools receive eight implementation strategies that effectively support policy implementation (highest potential for policy implementation)

A complete description of PACE, including theoretical underpinnings, intervention design, and a comprehensive description of strategies, is published elsewhere [35, 37, 53]. Briefly, the multi-strategy intervention (PACE) was designed for delivery by the Local Health District, to support schools to implement the physical activity policy [58]. Each PACE strategy was determined, using a rigorous mapping process informed by the Behaviour Change Wheel (BCW) [59] and Theoretical Domains Framework (TDF) [60], specifically to address identified barriers to schools’ implementation of physical activity policies [61]. Table 1 includes an overview of the final implementation strategies (described using the School Implementation Strategy taxonomy [62]): centralised technical assistance and ongoing consultation/coaching; principals mandating for change; identifying and preparing in-school champions; development of implementation plans; educational outreach visits; the provision of educational materials and change/alter the school environment [35, 37]. Schools randomised to receive PACE still had access to usual care (general resources, information, and reactive support from health service staff); however, the suite of PACE strategies were designed to replace the need for reactive support by providing consistent and ongoing support to all schools. A logic model depicting the different paths for PACE and usual care can be found in Fig. 1.

**Table 1:** A description of implementation strategies and the total cost to deliver each from the perspective of health service providers and schools

Strategy description and cost components*	Total cost (average per school)		
	Health service provider (base case)	Schools	Overall
1. Centralise technical assistance and provide ongoing consultation/coaching: project officers employed by the Local Health District provide expertise, advice, and resources throughout the study period. *Health service costs: labour (project officers)* *School costs: labour (classroom teachers)*	$3406 ($110)	$1772 ($57)	$5178 ($167)
2a. Mandate for change: 1 × initial meeting with school principal and school executives to communicate policy importance. *Health service costs: labour (project officers)* *School costs: labour (principals)*	$360 ($12)	$403 ($13)	$763 ($25)
2b. Mandate for change: schools either develop a physical activity policy or review/amend an existing one. *Health service costs: N/A* *School costs: labour (principals)*	$0 ($0)	$3343 ($108)	$3343 ($108)
3a. Identify champions: each school nominates 1–2 in-school champions. *No costs (incorporated within strategy 2a)*^*a*^	$0 ($0)	$0 ($0)	$0 ($0)
3b. Prepare champions: 1 × full-day training session for nominated in-school champions. *Health service costs: labour (project officers); materials; workshop expenses; travel and expenses* *School costs: N/A*	$19,437 ($627)	$0 ($0)	$19,437 ($627)
4. Develop a detailed implementation plan: in-school champions develop a plan for policy implementation in their school *No costs (incorporated within strategy 3b)*^*b*^	$0 ($0)	$0 ($0)	$0 ($0)
5a. Conduct educational outreach visits: 1 × 1–2 h teacher information session and training delivered during school staff meeting. *Health service costs: labour (project officers); travel and expenses* *School costs: labour (classroom teachers and principals)*	$4992 ($161)	$26,118 ($843)	$31,110 ($1004)
5b. Conduct educational outreach visits: if needed, 1 × 15 min follow-up teacher support session delivered during school staff meeting. *Health service costs: labour (project officers); travel and expenses* *School costs: labour (classroom teachers and principals)*	$1536 ($50)	$9839 ($317)	$11,375 ($367)
6a. Develop and distribute educational materials: ISC receive an “intervention manual” inclusive of policy templates as well as physical activity timetable and PE curriculum examples. *Health service costs: materials* *School costs: N/A*	$60 ($2)	$0 ($0)	$60 ($2)
6b. Develop and distribute educational materials: educational materials distributed to in-school champions and classroom teachers in print copy and accessible via an online portal. *Health service costs: materials* *School costs: N/A*	$2131 ($69)	$0 ($0)	$2131 ($69)
7. Capture and share local knowledge: case studies from other schools available via an online portal. *No costs (embedded within an existing online portal)*^*c*^	$0 ($0)	$0 ($0)	$0 ($0)
8a. Change/alter environment: each school provided with one physical activity pack (basic equipment). *Health service costs: materials* *School costs: N/A*	$3100 ($100)	$0 ($0)	$3100 ($100)
8b. Change/alter environment: project officers encourage in-school champions encouraged to develop physical activity packs for each classroom using existing school sport equipment. *Health service costs: labour (project officers)* *School costs: N/A*	$671 ($22)	$0 ($0)	$671 ($22)
Average cost per school	$1151 (95% UI $1046, $1236)	$1338 (95% UI $1048, $1663)	$2489 (95% UI $2130, $2832)

*Full details of the cost components are provided in Table 2

^a^Nomination of an in-school champion is an agenda item during the principal meeting, and thus the cost associated within this strategy is integrated within strategy 2a

^b^Time is allocated to develop a detailed implementation plan during the full-day training session for in-school champions, and thus the cost associated with this strategy is integrated within strategy 3b

^c^Case studies are available on an existing online portal with 'how to access' described during training sessions (strategies 3b and 5)

### Measurement of trial outcomes

The primary outcome was the mean minutes of physical activity implemented by classroom teachers across one school week, as recorded in a daily teacher log-book at baseline and 12-month follow-up. Teacher log books with less than one full week of data (< 5 days) or > 250 min scheduled across the school week were considered invalid and excluded from analysis. Secondary outcomes were the individual types of physical activity implemented by teachers that made up the primary outcome of mean minutes of physical activity: PE, energisers, sport, and integrated lessons.

### Identification, measurement and valuation of resource use

The focus of analysis was on the costs and cost-effectiveness of implementing PACE, therefore costs related to research or intervention development or start-up were excluded. All cost data were prospectively recorded in project management records maintained by project officers and research staff over the course of the trial. This included invoices/receipts of all expenses valued at market prices and records of each implementation activity that took place (facilitator, type, and duration). Cost data were used to calculate the following outcomes: total intervention cost, cost per strategy, and incremental cost. All cost-related outcomes were calculated overall across all schools as well as an average per school.

### Health service

Resource use by the health service for intervention delivery was prospectively identified and measured. Table 2 provides details of the four respective cost components: (i) labour—project officer time spent implementing strategies (midpoint wage rates); (ii) materials—printing and purchase of consumables (exact costs valued at market prices); (iii) workshop expenses—venue hire and catering (exact costs valued at market prices); and teacher relief to attend (i.e. reimbursement; midpoint wage rates); and (iv) travel and expenses—meals, travel and accommodation for project officers to deliver strategies (exact costs). Table 1 notes which cost components pertain to each strategy for both the health service and schools.

**Table 2:** Cost components attributed to Local Health District and schools as well as the details and sources of unit costs

Cost components	Detail	Source of unit costs
Local Health District:		
Labour - Support (via in-person, email or telephone) - In-person training	Project officers employed by the Local Health District, time to deliver the intervention	Project officer wage rates (Health Professional and Medical Salaries [State] Award 2019; Industrial Commission of New South Wales): Health Education Officer (mid-point) $48 per hour.
Materials - Print educational handouts for teachers - Physical resources for teachers (whistles and USB sticks) - Physical activity equipment - In-school champion manuals (print) and t-shirts	Printing costs and purchase of materials	Exact costs from project records; valued at market prices
Workshop expenses	a) Venue hire and catering b) Teacher relief to attend	a) Exact costs from project records; valued at market prices b) Teacher relief based on school staff wage rates (Crown Employees [Teachers in Schools and Related Employees] Salaries and Conditions Award 2019 –Industrial Commission of New South Wales): classroom teacher (mid-point) $44.57 per hour; school executive (mid-point) $53.75 per hour
Travel and expenses	Meals, travel, and accommodation costs	Exact costs from project records
Schools:		
Labour - Principal - In-school champion (a classroom teacher) - Classroom teacher	Time in hours, estimated by research staff using project records	School staff wages rates: (Crown Employees [Teachers in Schools and Related Employees] Salaries and Conditions Award 2019 –Industrial Commission of New South Wales): classroom teacher (mid-point) $44.57 per hour; school executive (mid-point) $53.75 per hour

Note: Data for each cost component were extracted from project management records maintained by project officers and research staff over the course of the trial

### Schools

For this trial, the only cost component of schools was labour, representing principal or classroom teacher time to implement strategies (midpoint wage rages) (Table 2). Using the project management records, project staff estimated the costs incurred by each school who received the intervention in the form of staff time (hours) taken to implement the intervention. This included: in-school champion or principal engagement with project officers (based on records of each communication), staff training (based on recorded duration and number of attendees), or working on their school physical activity policy (assumed to be 1.5 h based on feedback from in-school champions). We did not ask schools to report this level of information themselves as it was considered subsidiary data and to prevent overburdening school staff [63].

### Economic analyses

All analyses were carried out using Microsoft Excel software 2013. The base case analysis was undertaken from the health service perspective—the perspective most relevant to decision makers and funders of PACE, and the most practical and appropriate approach in the context of the research trial. However, incorporating a societal perspective would capture consequences more broadly [64]; thus we addressed this by way of a sensitivity analysis (details reported below). The total intervention costs and cost per strategy were calculated and as an average per school. In the base case analysis we assumed that the intervention costs were wholly incremental to usual care. Finally, ICERs were calculated by dividing the incremental cost by the estimated intervention effect of PACE. ICER values were calculated using paired school level cost and outcome data. The ICER represents the additional cost to achieve an additional minute of physical activity implemented per school week as a result of receiving PACE, per school. A cost consequence analysis was undertaken as a secondary analysis, presenting the average cost per school associated with the delivery of PACE alongside the treatment effects for the individual components of physical activity scheduling that sum to create the primary outcome, minutes of overall weekly physical activity scheduled. The individual components assessed included PE, energisers, sport, and integrated lessons.

### Sensitivity analyses

Three, one-way sensitivity analyses were undertaken to evaluate the robustness of the results from the base case analysis, when applying different assumptions in the estimation of a number of cost and outcome parameters.

The first one-way sensitivity analysis was undertaken to explore the cost effectiveness of PACE from a societal perspective, by incorporating the estimated school related costs into the calculated cost of delivering the intervention. In the base case analysis, we took a health service perspective, assessing only health service-related costs (most relevant for the study purpose). For this first sensitivity analysis, school-level costs incurred from PACE were estimated by the team (described above for ‘Schools’ under ‘Identification, measurement, and valuation of resource use’ sections) as we were unable to collect this information directly from schools due to resource restraints and to reduce burden on schools.

The second one-way sensitivity analysis was conducted by adjusting the assumed health service-related cost of delivering PACE in relation to the current cost of usual care. In the base case analysis, we assumed that the cost of PACE was wholly incremental to the cost incurred by usual care, as intervention schools still technically had access to all components of usual care. This is likely an overestimation of the cost incurred by the health service in the delivery of PACE, as PACE strategies were designed to replace the current components of usual care related to supporting school compliance with the physical activity policy. However, usual care does not focus solely on supporting schools to comply with the policy, but with a range of obesity prevention programs. Consequently, it was not possible to disentangle the costs associated with the usual care support provided for the policy from the range of other programs. At the time of this study, a dedicated cost of AUD$700 per school per year is allocated to cover the support provided to schools for these obesity related programs, including the physical activity policy. As PACE was designed to replace usual care this sensitivity analysis assumes a usual care cost of $700 per school, which is incorporated into the cost of delivering PACE.

The final one-way sensitivity analysis used an alternate approach to imputing missing outcome values for schools without valid 12-month follow-up data. As only a small number of schools had missing follow-up data (three intervention and one control) we used a last observation carried forward imputation for the base case analysis. For the third sensitivity analysis we employed mean imputation to account for these missing values.

### Scenario analyses

Two scenario analyses were undertaken to assess potential impacts on the cost-effectiveness of PACE when altering strategies identified as the largest drivers of intervention cost (i.e. in-person training and facilitation by trained project officers). Specifically, these scenario-based analyses assessed the proposed impact of optimising the PACE intervention by adapting the most expensive intervention components to less costly modes of delivery. Adjustments to the effect size were made based on current evidence, to account for the commonly observed scale-up penalty that occurs once interventions are adapted and scaled [65]. Table 3 describes the scenarios, the assumptions made, and the justification for these assumptions.

**Table 3:** Possible scenarios (‘best’ and ‘worst’ case) for scaling up PACE

Scenario	Description	Assumption and justification
A. Best-case scenario: Scale-up removing in-person strategies with best-case effect	Strategy 3b is removed. Strategy 5a and 5b are removed as a health service delivered strategy, and assumed to be performed by the in-school champion as part of their program role. The effect size has been reduced using a scale-up penalty based on a recent review. In this analysis, it is assumed that 60% of the original treatment effect is maintained. Specifically, the estimated average treatment effect and lower and upper intervals were each reduced by 40% to obtain a re-calculated treatment effect based on scale-up penalty that maintains 60% of the original effect size.	It is assumed that delivery of the in-person training is unfeasible for wide scale-up. An alternate version of PACE is proposed whereby in-school champions and teachers receive training via a self-directed online course. This course is envisaged to be integrated as part of teachers continuing professional education, thus no additional costs are assumed. The team are currently developing this optimised iteration of PACE. Systematic review evidence determined that the effect estimates from scale-up of effective interventions are reduced. However, the extent to which the estimated effects are reduced is variable. For this scenario, we adjusted the effect size based on a recent review finding that physical activity interventions delivered in community settings (e.g. schools) maintain a median 60% of the original effect size [65].
B. Worst-case scenario: scale-up removing in-person strategies with worst case effect	A replication of Version A assuming a worst-case scale-up penalty of only 25% of the original effect size is maintained. Specifically, in this scenario the estimated average treatment effect and lower and upper intervals were each reduced by 75% to obtain a re-calculated treatment effect based on a scale-up penalty that maintains 25% of the original effect size.	Systematic review evidence determined that the effect estimates from scale-up of effective interventions are reduced. However, the extent to which the estimated effects are reduced is variable. This scenario provides the worst case scenario in terms of possible scale-up penalty, with the effect size adjusted based on the lower end of the scale-up penalty values found in the recent review [65].

### Uncertainty and handling of missing data

To account for sampling variation, bootstrapping analysis with 1000 replications were undertaken to calculate uncertainty intervals (UI) around each of the main outcomes. Data quality monitoring processes were undertaken to identify and address missing cost data prior to analysis; any missing data was resolved through consultation with the project officer responsible for the missing values. Missing school-level outcome data at 12-month follow-up was imputed using the last observation carried forward for the base case analysis.

## Results

### Trial outcomes

Trial outcomes have been published separately [37]. In summary, the trial included 400 teachers (221 intervention, 179 control) at baseline, and 403 teachers from 57 schools at 12-month follow-up who provided valid outcome data. Table 4 outlines the characteristics of the base case population (i.e. the teachers in the sample). On average, classroom teachers at follow-up were 39 years of age and had 13 years teaching experience; the majority were female (86%) and employed full-time (87%).

**Table 4:** Teacher characteristics by experimental group

Characteristic	Control		Intervention	
	Baseline	12-months	Baseline	12-months
School type teaching at	*N* = 179	*N* = 180	*N* = 221	*N* = 223
• Catholic/independent	62 (35%)	72 (40%)	66 (30%)	67 (30%)
• Government	117 (65%)	108 (60%)	155 (70%)	156 (70%)
Age of class teacher	*N* = 173	*N* = 158	*N* = 202	*N* = 197
• Mean (SD)	38.0 (11.1)	38.3 (11)	40.0 (11)	39.8 (11)
Sex	*N* = 174	*N* = 175	*N* = 210	*N* = 219
• Female–*n* (%)	148 (85%)	149 (85%)	183 (87%)	189 (86%)
Job share	*N* = 173	*N* = 168	*N* = 209	*N* = 211
• Yes–*n* (%)	53 (31%)	48 (29%)	48 (22%)	49 (23%)
Employment status	*N* = 172	*N* = 170	*N* = 209	*N* = 209
• Permanent full-time	104 (60%)	88 (52%)	113 (54%)	111 (53%)
• Temporary full-time	50 (29%)	62 (36%)	71 (34%)	67 (32%)
• Permanent part-time	7 (4%)	11 (6%)	14 (7%)	15 (7%)
• Temporary part-time	6 (3%)	5 (3%)	8 (4%)	13 (6%)
• Casual	5 (3%)	4 (2%)	3 (1%)	3 (1%)
Number of years teaching	*N* = 172	*N* = 167	*N* = 209	*N* = 207
• Mean (SD)	13.0 (11)	12.5 (10)	14.6 (10)	13.8 (10)
Specialist PDHPE teacher	*N* = 173	*N* = 168	*N* = 211	*N* = 210
• Yes–*n* (%)	3 (2%)	0 (0%)	2 (1%)	6 (3%)

### Intervention costs

All 31 intervention schools were included in the economic analysis with complete cost data for each. Table 1 includes an overview of calculated costs of PACE and each discrete strategy. For completeness and transparency we have listed all strategies included in PACE, even those with no associated cost. From the health service perspective (base case analysis), the total cost to deliver PACE was calculated to be $35,692 (95% UI $32,411, $38,331). The average cost per school was calculated to be $1151 (95% UI $1046, $1236) (Table 1). No costs were associated with strategies 3a (identify champions), 4 (develop a detailed implementation plan), and 7 (capture and share local knowledge) as these were integrated within other strategies. Strategy 3b (full-day training session for nominated in-school champions) was the largest contributor to total cost, calculated to be $19,437 overall and $627 ($0 to $648) per school. Strategy 5b (1–2 h teacher training and information session) was the second largest contributor to total cost, calculated to be a total of $4992 to deliver for an average of $161 ($0 to $528) per school.

### Cost-effectiveness analysis

**Fig. 2:**
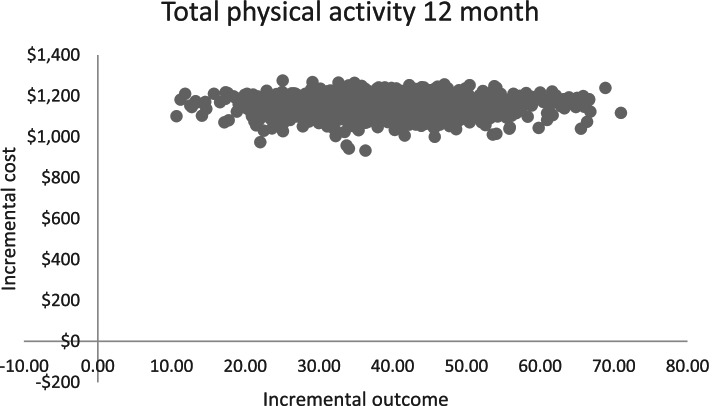
Bootstrapped scatterplot of the incremental cost-effectiveness ratio (ICER) values for the primary outcome (total minutes of physical activity) displayed on the cost-effectiveness plane

The ICER when accounting for the costs incurred by the health service only (base case analysis) was $29 (95% UI $17, $64) for every additional minute of weekly physical activity implemented per school (Table 5). The joint distribution of the primary outcome of minutes of physical activity implemented and cost from the 1000 bootstrapped replications are shown on the cost-effectiveness plane in Fig. 2. All values fall within quadrant two of the cost-effectiveness plane, indicating that PACE is a more effective intervention than usual care but at a higher cost.

**Table 5:** Incremental cost-effectiveness ratio (ICER) values and cost-consequence analysis

Cost incurred by:	Total cost per school (95% uncertainty interval)
**The health service provider** *(primary analysis)*	$1151 ($1046, $1236)
Schools *(sensitivity analysis)*	$1338 ($1048, $1663)
Overall	$2489 ($2130, $2832)
**Implementation outcome–total weekly minutes of**	**Mean difference in change in outcome at 12-months (95% confidence interval)**
PE	10.4 (1.89, 18.8)
Energisers	23.1 (16.5, 29.6)
Sport	3.81 (− 3.13, 10.8)
Integrated lessons	6.96 (3.15, 10.8)

*Note*: Comparator costs assumed to be $0 for base-case analysis

### Sensitivity analyses

**Fig. 3:**
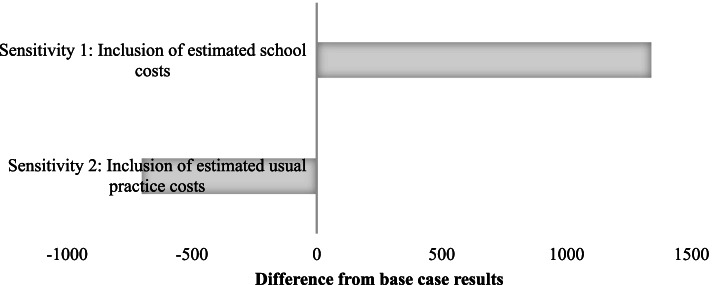
Results from the sensitivity analyses illustrating the difference in the average incremental costs per school between the base case analysis and the sensitivity analyses. The top bar shows the results relating to the first sensitivity analysis whereby the estimated costs associated with schools were included in the intervention costs. The bottom bar shows the results relating to the second sensitivity analysis whereby the cost incurred by schools who received usual support was assumed to be $700

The first sensitivity analysis took a societal perspective, whereby the estimated school associated costs were included in the intervention costs. For this analysis, the total estimated school-level cost was $41,474 (95% UI $32,489, $51,541), or an average of $1338 (95% UI $1048, $1663) per school. Combining the estimated school levels costs with the health service costs, PACE cost an estimated total of $77,166 (95% UI $66,018, $87,778) to deliver, or $2489 (95% UI $2130, $2832) per school. Compared to the base case analysis, this represented almost twice the estimated cost associated with intervention delivery (Fig. 3). The calculated ICER was $63 (95% UI $35, $140) for every additional minute of weekly physical activity implemented per school (Table 5 and Fig. 3).

For the second sensitivity analysis whereby the cost of usual practice was assumed to be $700 per school and was incorporated into the cost of delivering PACE, the total incremental cost was $14,692 (95% UI $11,601, $17,004) and the average incremental cost per school was $451 (95% UI $352, $526). Compared to the base case analysis, this represented almost half the estimated cost associated with intervention delivery (Fig. 3). The calculated ICER was $11 (95% UI $6, $28) for every additional minute of weekly physical activity implemented per school (Fig. 3).

The final one-way sensitivity analysis used mean imputation to address missing outcome data at 12-month follow-up for four schools (three intervention and one control). In this analysis, the cost outcomes do not change as the cost data is consistent with the base case; however, the calculated ICER, incorporating the differences in the outcome data, is $27 (95% UI $17, $50) for every additional minute of weekly physical activity implemented per school.

### Cost consequence analysis

Table 5 displays the different costs incurred by the organisations involved in delivering PACE as well as overall, alongside the estimated effects for the different components of physical activity that combine to create the primary outcome. PACE had the greatest effect on energisers and the least impact on the number of weekly minutes scheduled for sport.

### Scenario analyses

#### Scenario A: best-case

If we were to replace the strategies with the largest cost (strategy 5 and strategy 3b; due to in-person training and facilitation by trained project officers) with no-cost alternatives (i.e. delivery via in-school champion and online learning), and were able to maintain 60% of the original treatment effect (Table 3), it would cost approximately $13 (95% UI $8, $29) for every additional minute of weekly physical activity scheduled per school.

#### Scenario B: worst-case

**Fig. 4:**
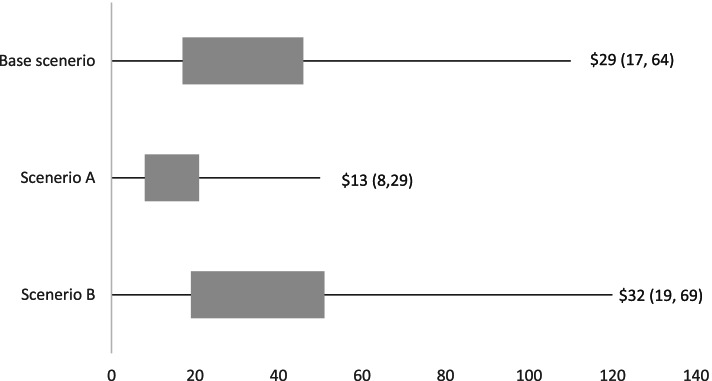
Incremental cost effectiveness ratio (ICER) values and 95% uncertainty intervals (UI) for the base case (top bar) and scenario analyses (following two bars). The middle bar represents scenario (**A**): replacing the 1-day training session for in-school-champions with a no-cost online educational session and maintaining 60% of the original treatment effect (‘best case’ scale-up penalty). The bottom bar represents scenario (**B**): replacing the 1-day in-school champion training session with a no-cost online educational session and maintaining 25% of the original treatment effect (‘worst case’ scale-up penalty)

With these same strategy amendments, if the treatment effect dropped to 25% of the original effect (Table 3) the cost for every additional minute of weekly physical activity scheduled per school is estimated at $32 (95% UI $19, $69). Figure 4 displays a comparison of the scenario based cost effectiveness estimates compared to those obtained from the primary analysis.

## Discussion

This study is one of few that have examined the costs and cost-effectiveness of public health implementation interventions in the school setting [41]. Overall, PACE cost an estimated total of $77,166 (average of $2489 per school), with $35,692 ($1151 per school) incurred by the health service to deliver the intervention. This is important information for decision-makers of the investment required to support schools to implement a mandated physical activity policy and consequently, to achieve the intended benefits of this evidence-based practice. The average cost of each discrete PACE implementation strategy ranged from $0 to $627 per school. One of the strengths of this evaluation is the ability to identify the more costly strategies where adaptations might be targeted to reduce the total cost, thus improving its potential for scale-up. The ICER was calculated at $29 (95% UI $17, $64) for every additional minute of weekly physical activity implemented per school. While there is limited guidance of what is considered cost effective for implementation outcomes (such as in this study), when considered in relation to the available literature these findings do suggest the potential of PACE to be a cost-effective intervention for increasing school implementation of a policy mandate. This is discussed in relation to the literature following.

Due to the limited CEA of implementation strategies in the school setting [41] and given that none have explored physical activity policy implementation, we were unable to identify any explicit willingness to pay threshold for minutes of physical activity provided by teachers. Thus, to gauge the possible cost implications of PACE, we interpreted the CEA within the context of the results from similar interventions delivered by health service providers [66], and to Local Health District’s likely willingness to pay to support schools implementation of physical activity programs.

### Findings from similar studies

We identified only three economic evaluations of school-based physical activity implementation strategies, two of which assessed outcomes at the individual-level rather than implementation [44, 67]. The other, including a measure of implementation, a 2021 CEA of a package of implementation strategies (*n* = 7) designed to assist secondary schools to implement an evidence-based program for increasing adolescents physical activity [45]. The study adopted a public finance perspective by incorporating costs incurred by the health service and schools. The calculated ICER was reported as $25,944 per percent increase in the proportion of schools implementing ≥ 4 of the seven strategies. It is difficult to interpret this ICER in relation to our own without a comparable outcome measure, and without disentangling the costs incurred by the health service provider and schools. Of interest, the evidence-based intervention itself had previously been deemed cost effective with an ICER of AUD$56 per each additional minute of moderate-to-vigorous physical activity gained per day [44]. Although assessed in minutes of physical activity, this is also difficult to compare with our ICER which used weekly minutes and obtained from teacher's schedules rather than objective student accelerometer data. Regardless, inferential reasoning would suggest that $56 per minute daily, when considered across a 5-day school week, is higher than our ICER of $29 per weekly minute.

We are also able to draw on a relevant study from the field of nutrition: a 2018 CEA of a multi-strategy support intervention to enhance schools’ implementation of a food availability policy in Australia [66]. An ICER for the intervention, delivered by the Local Health District at three different intensities (high, medium, and low), was calculated as the incremental cost per additional percentage point increase in proportion of schools reporting policy adherence. The medium intensity intervention was emphasised as the superior option for delivery at-scale by the health service, with an ICER of AUD$2627 (versus AUD$2982 for high and AUD$4730 for low). In the PACE RCT, intervention schools were, on average, 35 min from policy compliance. Crudely estimated, our ICER of AUD$29 per minute increase in minutes of physical activity scheduled equates to an approximate average of AUD$1015 per school to reach policy compliance, which is a considerably lower investment than what was accepted by the same health service as reasonable to spend in supporting schools to comply with a nutrition-related policy.

### Health service provider’s willingness to pay

Interpreting the cost-effectiveness of PACE in this study requires consideration of what the health service provider is willing to invest for schools’ implementation of the physical activity policy. The Local Health District, which is the health service provider in this instance, is already funding $700 per school per year to assist schools to improve the health of students via ‘usual care’ practices. Our baseline data showed that this approach has not facilitated implementation of the physical activity policy by schools [37]. Some investment is required by the health service provider to make use of the existing funding and infrastructure which, without PACE, does not adequately improve policy implementation. The sensitivity analysis that incorporates usual care costs shows how the additional cost above that already provided may not actually be that much.

Another consideration for the health service provider, when deliberating the value of investment in PACE, is the return on investment resulting from increased implementation of the policy (an evidence-based practice) [43]. Specifically, increased policy compliance by schools in this study has the potential to improve child physical activity [12, 35, 68] and consequently positively impact on their physical, physiological and cognitive health [1]. In turn, and especially when achieved at a population-level, such health improvements reduce healthcare costs. The inverse relationship between school-based physical activity and healthcare costs is supported by research. For example, micro-simulation models of interventions to increase the duration and/or quality of physical activity provided by schools have shown substantial estimates for healthcare savings in several high-income countries due to avoidance of direct healthcare costs related to the treatment and management of disease [21]. In Australia, an AUD$10 million investment in school-based interventions to increase physical activity over 6 years showed healthcare savings of AUD$641 million [22]. In Canada, each CAD$100 per student spent on programs to increase the duration and quality of PE showed healthcare savings of CAD$484 [21]. Finally, in the USA, similar PE policies over the course of 6 years showed healthcare savings of US dollars [USD]$35.4 million and an estimated 13,652 cases of childhood obesity prevented [69]. Even greater benefits were shown with policies increasing school day physical activity to ensure 150 weekly minutes (like PACE), with USD$171 million in healthcare savings and 73,589 cases of childhood obesity prevented. The advantages of implementing the school day physical activity policy compared with the PE policy were due to an increased reach of students in the first year (31.3 million students vs. 21.7 million students) [69].

### Energisers: a key area of future investment

The cost consequence analysis provides decision-makers with interpretable findings relating to the individual types of physical activity that collectively formed the primary outcome of total physical activity. In the RCT, PACE obtained most of its treatment effect from energisers and the cost consequence analysis performed in the current study showed that this was at a minimal cost to the health service provider. A 2014 economic analysis comparing school-based approaches to increase physical activity found that two of the four types of programs were significantly superior in terms of reach and cost per student—one being short physical activity breaks, i.e. energisers during class time [70]. Energisers may be a key area of investment for future delivery of PACE, to achieve policy implementation by schools in a cost-efficient manner.

### Cost implications of scale-up

Implementation interventions must be feasible and low cost for delivery at scale [71]. We are aware that some of the more intensive PACE strategies, particularly those delivered in-person, although effective in supporting policy implementation, may not meet these requirements. Adaptations are common of physical activity interventions as they transition from research settings to broader dissemination [65]. We performed scenario analyses to explore the potential impact of adapting more costly strategies to improve not only intervention feasibility, but also the investment required by the health service provider to deliver PACE at scale. The most cost-effective scenario was the PACE variation with all in-person support removed and sustaining the lowest possible scale-up penalty (‘best case’ scenario from systematic review evidence = 40% drop in effect size [65]). The ICER for this ‘best case’ scenario was less than half of that calculated for the current PACE intervention ($13 vs $29). However, a more severe scale-up penalty is possible (‘worst case’ scenario from systematic review evidence = 75% drop in effect size [65]). While our analysis showed that this ‘worst case’ scenario was less cost-effective, the investment for each minute increase in implemented physical activity was on average only $3 more than the current PACE intervention ($32 vs $29). Moreover, we are confident that the scale-up penalty sustained by removing in-person support (training school stakeholders via self-directed online rather than in-person by a project officer) would be minimal; i.e. close to the ‘best case’ scenario. Evidence shows that online professional development is effective [72]; it has become more normalised in the COVID-19 pandemic; and it provides advantages for teachers such as flexibility, convenience, a self-paced format and ability to be delivered to all teachers rather than just a select sample of in-school champions [73]. However, it also has potential disadvantages for teachers such as lack of personalisation and limited opportunities for collaboration [73]. An online PACE delivery platform would benefit from comprehensive formative work to ensure that this approach retains a meaningful impact. As a starting point, it may be informed by a recent systematic review of elements related to the design and delivery of effective online professional development programs for teachers (e.g. engagement, flexibility, accommodating different learning styles and including practical learning activities) [74].

## Limitations

The findings of this study should be interpreted in light of several limitations. First, it is difficult to interpret the ICER without an explicit willingness to pay threshold however the translation of teachers’ scheduled minutes of physical activity into a more comparable, clinical outcome (e.g. BMI or quality adjusted life years [QALY]) was beyond the scope of this economic evaluation. More work is needed to determine what is meaningful in terms of cost-effectiveness for implementation outcomes to allow for more informed assessments of ICER related values in this field. Second, consistent with our a priori economic evaluation plan, we included sensitivity tests that were considered to be of the greatest value to decision makers; however, there are other potential deterministic sensitivity tests worthy of future exploration which were outside the scope of the current study (e.g. including variation in the role and hence value of the labour time). Third, although we used a prospective design, there were some instances in which we had to retrospectively collect cost-data, when data was missing and follow-up with project officers was required. We are currently improving the protocol to address this for future trials. Third, school level data were collected by research staff and may not be an accurate representation of the costs incurred by schools. It was also difficult to determine whether the opportunity cost of staff time for the training session was attributable to PACE as it took place during a regularly scheduled staff meeting. In fact, cost-simulation of physical activity policy implementation in schools has estimated only 3% of total costs are incurred by schools [69]*.* Such findings suggest that the results found from our base case analysis are likely a minimal underestimate of the cost of the intervention compared to the sensitivity analysis that incorporated the estimated school related costs. Future evaluations should consider more efficient ways of capturing costs from the school perspective. Finally, the cost and ICER values are likely to be an overestimation due to a lack of a reliable comparator and use of school level outcomes compared to the more precise teacher level outcomes modelled using sophisticated statistical modelling used in the primary evaluation of the trial. More reliable methods of collecting comparator cost data from control schools that does not produce excessive burden on schools is needed.

## Conclusion

Adoption of PACE as the new standard health service delivery intervention requires consideration of not only intervention effectiveness but also what the cost is and whether it is worth the investment required by the health service provider. We found that PACE is a potentially cost-effective means of increasing primary schools’ compliance with an Australian state mandate for school day physical activity, based on the investment the health service has been willing to make to support schools' compliance with other obesity related programs in the past. Adaptations may be made to substantially improve the cost required by the health service provider to deliver PACE, without compromising the intervention cost-effectiveness—specifically, training delivered via an online learning platform.

## Data Availability

The datasets used for the current study are available from the corresponding author upon reasonable request.

## Abbreviations

WHO: World Health Organization
PE: Physical education
CAD: Canadian dollar
RCT: Randomised controlled trial
CEA: Cost-effectiveness analysis
BMI: Body mass index
PACE: Physically Active Children in Education
NSW: New South Wales
AUD: Australian dollars
ICER: Incremental cost-effectiveness ratio
BCW: Behaviour Change Wheel
TDF: Theoretical Domain Framework
UI: Uncertainty interval
USD: US dollar
QALY: Quality-adjusted life year
